# Recent Advances in Pathogenicity and Biocontrol of Postharvest *Penicillium* Diseases

**DOI:** 10.3390/jof12030219

**Published:** 2026-03-18

**Authors:** Guohua Yin, Siyuan Zhao, Han Zhang, Kayla K. Pennerman, Joan W. Bennett

**Affiliations:** 1 College of Biological and Chemical Engineering, Qilu Institute of Technology, Jinan 250200, China; 2 School of Natural Sciences & Mathematics, Stockton University, Galloway, NJ 08205, USA; 3 Department of Ecology and Evolutionary Biology, Tulane University, New Orleans, LA 70118, USA

**Keywords:** *Penicillium*, postharvest disease, CRISPR-Cas9, RNA interference, sustainable control

## Abstract

*Penicillium* species are major postharvest pathogens of fruits and vegetables, causing significant economic losses and posing serious threats to food safety through mycotoxin contamination. This review systematically summarizes the pathogenic mechanisms, metabolic diversity, and eco-friendly strategies of postharvest *Penicillium* pathogens. The application of CRISPR-Cas9 technology has enabled precise functional analysis of pathogenicity-related genes (e.g., *PacC*, *PeStuA*) and regulatory elements involved in fungicide resistance (e.g., *FlbC*). RNA interference-based strategies, including host-induced gene silencing (HIGS) and spray-induced gene silencing (SIGS), offer promising non-transgenic approaches for disease control. Additionally, artificial intelligence-assisted species identification and fermentation regulation have improved research efficiency. Future integration of multidisciplinary technologies will facilitate sustainable management of postharvest diseases.

## 1. Introduction

The genus *Penicillium* comprises a group of saprophytic fungi widely distributed in nature, commonly found in diverse environments such as soil, plant residues, and even deep-sea sediments. This genus encompasses over 300 species, many of which possess complex metabolic pathways yielding bioactive compounds with significant value. Metabolites from *Penicillium* strains demonstrate substantial practical implications in drug development, food safety, and agriculture [1]. However, they can also have known or potential toxicity [2,3]. Most *Penicillium* strains have been isolated from terrestrial soil. Although marine-derived strains are fewer in number, their unique living environments often lead to the production of structurally novel metabolites, making them an important source for discovering new compounds [4].

Beyond their ability to synthesize valuable metabolites, certain *Penicillium* species are responsible for severe plant infections in major fruits (such as citrus, apples, pears, and grapes) and vegetables (including onions, garlic, and tomatoes), leading to substantial yield losses. Postharvest blue mold in citrus and apples caused by *P. digitatum* and *P. expansum* represents a global challenge. These pathogens not only induce fruit rot and the emission of organic volatile compounds [3,5], but *P. expansum* also produces the mycotoxin patulin, posing a serious risk to food safety [2,6]. A thorough understanding of their pathogenic mechanisms is essential for developing effective and sustainable control strategies. Traditional genetic manipulation techniques, such as *Agrobacterium tumefaciens*-mediated transformation (ATMT), have limited efficiency, which has constrained functional genomics research in these fungi. The CRISPR-Cas9 system, known for its high efficiency and precision, has emerged as a revolutionary tool for reverse genetics in filamentous fungi [7]. In recent years, the successful application of this technology in both *P. digitatum* and *P. expansum* has enabled the systematic functional characterization of several key genes involved in pathogenicity and fungicide resistance [8,9].

Current strategies for managing fungal diseases in plants primarily rely on field management, chemical fungicide application, resistance breeding, and the development of transgenic plants. The long-term use of conventional fungicides can lead to pathogen resistance and human health issues related to pesticide residues. In the United States, *Penicillium* spp. causing apple bule mold have developed resistance to the fungicide difenoconazole through global transcriptomic changes, including the upregulation of cytochrome P450 monooxygenases and active efflux pumps [10]. Brazilian citrus pathogens have developed considerable resistance to multiple chemical classes, including azoles and benzodioxoles, prompting the exploration of alternatives like “killer” yeasts, essential oils and antimicrobial volatile substances [11]. A 2025 study in Moroccan citrus packinghouses revealed that high resistance rates to thiabendazole (61.3%) and imazalil (58.1%) among *P. digitatum* and *P. italicum* isolates, with some exhibiting dual resistance that compromises conventional control programs [12]. Traditional resistance breeding methods are frequently constrained by extended selection cycles and evolution of new resistance mechanisms. Meanwhile, the short-term commercial release and widespread adoption of transgenic plants face considerable challenges such as public acceptance [13]. These global cases underscore how resistance mutations and adaptive mechanisms necessitate integrated, eco-friendly management strategies, including generally regarded as safe (GRAS) salts and biocontrol agents, to ensure sustainable production and export compliance. Achieving sustainable control of fungal plant diseases is of great significance for ensuring food security and promoting sustainable agricultural development. This review aims to systematically outline the study and control of postharvest diseases caused by *Penicillium* species. It focuses on the identification of pathogenicity and resistance-related genes in *Penicillium*, as well as the latest advancements in RNA interference (RNAi)-based strategies for its control, intending to provide a comprehensive reference for related research.

## 2. Systematic Analyses of the Function of Pathogenic Genes

### 2.1. Methodological Tools Advanced Understanding of Penicillium Pathogenicity

Significant progress has been made in elucidating the genetic basis of pathogenicity in *Penicillium* species, driven largely by advances in genomics and functional analysis tools [6]. *Agrobacterium tumefaciens*-mediated transformation has been successfully optimized in *Penicillium expansum* [14] and *P. italicum* [15]. This enabled the construction of mutant libraries and identification of novel pathogenicity determinants such as *PiBla* and *PiFTF1* in *P. italicum*, which regulate conidiation and virulence. More recently, RNA-based approaches have uncovered cross-kingdom RNA interference mechanisms in *P. italicum*, where silencing the Dicer-like gene Pit-DCL2 reduced fungal virulence and disrupted the biogenesis of microRNA-like small RNAs predicted to target host immunity genes [16]. Additionally, metabolomics-guided functional genetics revealed that the nonribosomal peptide synthetase HcpA is required for producing cyclic peptides (e.g., fungisporin) that act as time-regulated virulence factors during citrus infection [17].

The cell wall structure of *Penicillium* species poses significant challenges for genetic transformation. In 2022, Garrigues et al. conducted pioneering work by successfully establishing an efficient CRISPR-Cas9 system for both *P. digitatum* and *P. expansum* [7]. This study developed new, optimized protocols for protoplast preparation and transformation, utilizing a self-replicating plasmid based on the AMA1 backbone. A key advantage of this plasmid system is its recyclability—after gene editing is complete, the plasmid can be eliminated, enabling marker-free editing and allowing sequential rounds of genetic manipulation within the same strain. The authors validated the system by targeting the *wetA* gene, which regulates spore development, successfully obtaining mutant strains. These mutants exhibited reduced growth rates, sporulation defects, and altered pathogenicity [7]. While CRISPR-Cas9 is presented as a forward-looking functional genomics tool, most characterized genes to date were elucidated through *A. tumefaciens*-mediated transformation [14,18] and targeted gene replacement [19]. In the future, extensions of new CRISPR-Cas9 strategies will provide versatile and powerful platforms for precise, large-scale functional gene studies in pathogenic *Penicillium* species. Collectively, multi-faceted approaches—spanning genomics, ATMT, RNAi, CRISPR, and metabolomics—have substantially advanced the understanding of molecular mechanisms underlying *Penicillium* pathogenicity. The major pathogenicity-related genes and their functions are summarized in Table 1.

### 2.2. Penicillium digitatum

Using CRISPR and earlier ATMT techniques, a series of genes critical for the virulence of *P. digitatum* have been functionally validated. The pH-responsive transcription factor PacC functions as a global virulence regulator, and its deletion results in complete loss of pathogenicity, confirming that the pathogen adapts to the host microenvironment through pH modulation [20]. Deletion of the chitin synthase gene *PdChsVII* and the O-mannosyltransferase gene *Pmt2* severely compromises cell wall integrity, thereby impairing fungal growth and infection capacity, highlighting the essential role of cell wall biogenesis in pathogenicity [21,22]. Mutation of *PdMpkB* (a homolog of *Fus3/Kss1*) in the MAPK signaling pathway renders the pathogen nearly incapable of causing fruit rot, demonstrating that this kinase is indispensable for infection [26]. Garrigues et al. (2020) identified a unique cysteine-rich anionic secreted protein named Sca [23]. Although this protein lacks direct antimicrobial activity, its overexpression or exogenous application significantly enhances the infection success of *P. digitatum* on citrus fruits. Mechanistically, Sca effectively counteracts the protective effects of antifungal proteins (such as AfpB and PeAfpA) produced by the host or applied exogenously. Sca represents a class of virulence-enhancing factors that do not participate directly in basal metabolism but rather function to fine-tune host interactions and improve infection efficiency [23]. Mutations in the major facilitator superfamily (MFS) transporter genes *PdMfs1* and *PdMfs2* lead to reduced virulence of *P. digitatum* on citrus fruits. These transporters may be involved in the secretion of toxic compounds, such as tryptoquialanines, contributing to pathogenicity [27,34].

Functional genomics studies have elucidated a sophisticated regulatory network governing *Penicillium* pathogenicity. The transcription factor *PdSte12* is essential for conidiation and virulence during citrus infection, acting as a regulator of transporter-encoding genes and sterol demethylases [35]. Similarly, the *PdSlt2* mitogen-activated protein kinase controls sporulation and infection and also serves as negative regulator of several transporter encoding genes, such as ATP-binding cassette (ABC) and MFS transporters [36]. Major facilitator superfamily transporters play multifaceted roles, with *PdMFS1* contributing to both fungicide resistance and virulence [37], while PdMFS2-5 display differential functions during pathogen-fruit interaction [38]. The sterol 14α-demethylase *PdCYP51B* enhances both demethylation inhibitor resistance and fungal virulence through promoter modifications [39]. The recently characterized *PdMFS6* transporter contributes to chemical susceptibility and infectivity [40]. Notably, the transcription factor *PdMut3* exerts negative regulation on virulence, as its deletion unexpectedly increased pathogenicity during citrus infection, revealing complex transcriptional control mechanisms [41].

### 2.3. Penicillium expansum

Significant progress has been made in elucidating the genetic basis of pathogenicity in *P. expansum* and *P. italicum*, driven largely by advances in genomics and functional analysis tools. The publication of foundational genome sequences for both species has provided crucial insights into their virulence mechanisms and host specificity [19]. The APSES family transcription factor *PeStuA* has been identified as a core regulatory hub that globally controls hyphal growth, asexual sporulation, virulence, and patulin biosynthesis in *P. epansum* [29]. The acetate transporter *PepatA* positively regulates sporulation and patulin accumulation by modulating acetate metabolism [30]. These findings establish a close link between fundamental developmental processes, secondary metabolism, and pathogenicity in this fungus [42].

Genomic analysis indicates that *P. expansum* possesses a complete patulin biosynthetic gene cluster comprising 15 genes (*PePatA* to *PePatO*). In contrast, *P. digitatum* and *P. italicum* lack this complete cluster, explaining why *P. expansum* produces patulin while other citrus-infecting species do not [6,19]. Functional studies have confirmed that *PePatL* and *PePatK* play key roles in patulin biosynthesis; however, patulin production is not directly associated with the virulence of *P. expansum* [19]. The genome of *P. expansum* (33.52 Mb) is larger than those of *P. digitatum* (~26 Mb) and *P. italicum* (28.99 Mb), and it contains a greater number of carbohydrate-active enzymes (CAZymes) and secondary metabolite biosynthetic gene clusters. This expanded genetic repertoire may contribute to the broader host range of *P. expansum* [19]. Notably, pectin-degrading enzymes specific to *P. expansum*, particularly those belonging to the GH78 family, may provide the molecular basis for its ability to infect diverse fruits such as apples and pears [19].

### 2.4. Penicillium italicum

The pathogenicity of *P. italicum* is governed by complex genetic networks involving signaling, epigenetic regulation, and structural integrity. The genes *PiCaMK1*, *PiSntB*, and *Piwsc1* are all critical nodes within these networks. Knocking out any one of them significantly impairs the fungus’s ability to grow, reproduce, and cause disease, making them ideal targets for RNAi technology. RNAi could be applied to “silence” these essential genes by spraying citrus fruit with double-stranded RNA (dsRNA) molecules designed specifically against their transcripts. For instance, dsRNA targeting *PiCaMK1* would disrupt calcium signaling, hindering growth and stress response [31]. Silencing *PiSntB* could trigger a carbon starvation response and autophagy [32], while targeting *Piwsc1* would compromise cell wall integrity, reducing spore germination and lesion formation [33]. Because these genes are fundamental to *P. italicum* and have no known counterparts in plants or mammals, an RNAi-based fungicide could offer a highly specific, sustainable, and environmentally friendly strategy for controlling postharvest blue mold.

### 2.5. New Mechanisms for Fungicide Resistance Regulation

With the widespread application of fungicides, fungicide resistance has become an increasingly serious challenge. CRISPR technology has facilitated the discovery of novel resistance mechanisms that are independent of target gene (e.g., *CYP51*) mutations. Xi et al. (2024) combined transcriptomic analysis with CRISPR-based gene knockout to identify the transcription factor FlbC as a key positive regulator of resistance to DMI fungicides, such as imazalil, in *P. digitatum* [24]. Interestingly, although Δ*FlbC* mutants exhibited hypersensitivity to imazalil, their intracellular ergosterol levels remained unchanged. This indicates that *FlbC* regulates a novel resistance signaling pathway independent of the ergosterol biosynthesis pathway [24]. Earlier studies had already shown that the sterol regulatory element-binding protein homologs PdSreA and PdSreB influence the sensitivity of *P. digitatum* to DMI fungicides by regulating the expression of multiple sterol biosynthesis genes, including *CYP51* [25]. Major facilitator superfamily transporters are key multidrug resistance determinants in *Penicillium*, functioning as drug-H^+^ antiporters that actively extrude fungicides. In *P. digitatum*, PdMFS1-6 confers differential resistance to multiple fungicide classes and link chemical sensitivity to virulence [38]. Multi-drug resistant strains of *P. expansum* constitutively overexpress PeMFS1-2, with transporters also facilitating patulin export [43]. ABC transporters are also commonly associated with fungicide resistance in fungi [44]. Together, these findings depict a complex fungicide resistance regulatory network involving multiple layers of transcription factors.

### 2.6. Future Research

The application of CRISPR-Cas9 technology has successfully advanced research on the biology of postharvest *Penicillium* pathogens from traditional single-gene functional validation into the era of systematic dissection of pathogenicity and resistance regulatory networks. Current studies have not only confirmed classical “virulence-essential genes,” such as *PacC* [20] and cell wall synthesis-related genes [21], but have also expanded the conceptual framework to include newly defined categories such as “virulence-enhancing factors” (e.g., Sca) [23] and “dedicated resistance regulators” (e.g., FlbC) [24]. These findings have enriched our understanding of the infection strategies employed by these pathogens.

Building upon these achievements, future research directions may include the following: (1) advanced CRISPR applications employing CRISPR interference and CRISPR activation for precise gene expression modulation, as well as conducting genome-wide loss-of-function screens to systematically identify all genes contributing to pathogenicity; (2) in-depth mechanistic studies for investigation key factors already identified (such as FlbC and Sca), to elucidate their downstream target genes, interacting proteins, and the complete signalling pathways in which they function; (3) translational research for developing fungicides with novel modes of action, using molecular targets from these newly discovered pathogenicity and resistance genes, designing gene silencing-based control agents, and breeding disease-resistant fruit and vegetable varieties [42].

## 3. Etiologies and Metabolites of *Penicillium* Postharvest Diseases

The ecological and biological characteristics of *Penicillium* species exhibit profound regional variation, particularly between tropical and temperate zones. Research indicates that *Penicillium* species are more prevalent in temperate regions, while they are relatively rare in arid tropical environments where *Aspergillus* molds tends to dominate [45]. This geographical distribution reflects fundamental differences in adaptation, physiology, and metabolic potential. Cold-adapted strains from polar and high-altitude regions demonstrate remarkable psychrotolerance, with some species like *P. svalbardense* showing optimal growth at 17–18 °C and enhanced production of secondary metabolites under cold stress [46]. Himalayan isolates exhibit polyextremophily, tolerating wide temperature ranges, high salinity, and extreme pH while showing enhanced sporulation at low temperatures [47]. Conversely, tropical strains from biodiverse hotspots like the Brazilian Atlantic Forest display distinct species assemblages and metabolic profiles adapted to warmer, competitive environments [48]. Notably, strains from anthropogenically altered Antarctic habitats produce more structurally diverse secondary metabolites than those from undisturbed sites, suggesting environmental pressure shapes metabolic capacity [49]. Marine-derived *Penicillium* spp. from different climatic zones also yield distinct bioactive compounds, with recent reviews documenting 177 cytotoxic metabolites from marine strains between 2018–2024 alone [4]. These regional variations have significant implications for understanding pathogenicity mechanisms, predicting fungal responses to climate change, and developing targeted control strategies across different climatic zones.

**Figure 1:**
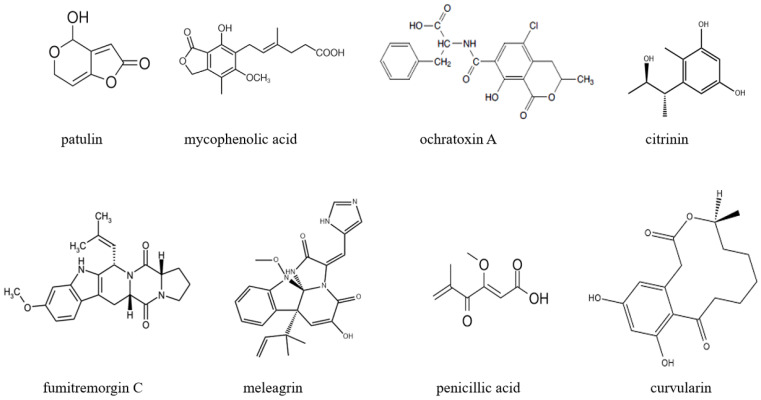
Chemical structures of some compounds biosynthesized by *Penicillium* species.

The primary species responsible for postharvest blue mold in apples include *P. digitatum*, *P. expansum*, and *P. italicum* [11,50,51]. During the infection process, *Penicillium* species generate a diverse array of secondary metabolites, including alkaloids, polyketides, terpenoids, and mycotoxins [52]. Many of these compounds exhibit a dual nature, possessing both toxic properties and beneficial bioactive potential [2]. The structures of some major *Penicillium* metabolites are presented in Figure 1.

### 3.1. Typical Mycotoxins

Produced primarily by *P. expansum*, patulin is the most significant mycotoxin associated with this species. It exhibits immunotoxicity, neurotoxicity, and potential carcinogenicity, making patulin a critical contaminant monitored in processed apple products such as juice and jam [2,6]. Strict regulatory limits have been established globally; the European Union sets a maximum limit of 50 μg/L for patulin in apple juice [53]. Produced by *Penicillium glabrum* IBRCM 30518, mycophenolic acid (MPA) possesses antiviral and immunosuppressive properties, while at high concentrations, it exhibits cytotoxic effects [54]. Produced by *Penicillium verrucosum* and *Penicullium nordicum*, ochratoxin A (OTA) is a mycotoxin known for its nephrotoxic and carcinogenic effects [55]. It is a contaminant of concern in fruits and their derived products [2]. Citrinin is produced by a number of species including *Penicillium citrinum* and *P. expansum* [50]. The compound has nephrotoxic properties and is frequently found together with ochratoxin A, with which it may exert synergistic toxic effects [2,56]. Fumitremorgin C is a neurotoxic mycotoxin previously reported in actinomycetes. Homologous compounds produced by *Penicillium* species are believed to have similar toxicological mechanisms and warrant further attention [57].

### 3.2. Alkaloids

Shentonins A, B, C, and D were isolated from *Penicillium shentong* XL-F41. Among them, shentonin D exhibits weak antibacterial activity against *Escherichia coli* (MIC = 100 μg/mL). Its nitrogen-containing heterocyclic structure is associated with toxicity modulation, warranting further investigation into its potential applications [58]. Derived from *Penicillium* sp. OUCMDZ-1435, meleagrin demonstrates anti-inflammatory potential. Because it exhibits cytotoxicity at high concentrations, additional studies will be needed to assess its therapeutic suitability [59]. Another classical alkaloid toxin, penicillic acid, displays both antimicrobial activity and cytotoxicity in mammalian cells, highlighting its dual biological effects [60].

### 3.3. Polyketone Active Ingredients

Azaphilone derivatives such as isochromophilone XV and arvoredol, isolated from *P. glabrum* SF-61290, exhibit significant cytotoxic activity against colorectal cancer HCT-8 cells, with efficacy surpassing that of the standard chemotherapeutic agent 5-fluorouracil [61]. Curvularin derivatives belonging to the curvularin family, including dehydrocurvularin, demonstrate marked inhibitory effects on the proliferation of HCT116 colorectal cancer cells [62].

### 3.4. Other Metabolites

Fatty acids and terpenoids derived from *P. shentong* XL-F41 exhibit potential metabolic toxicity, whereas, sesquiterpenoid toxins produced by *P. glabrum* demonstrate both antimicrobial activity and cytotoxic effects [58]. Macrolide compounds, such as brefeldin A derivatives, have shown promising bioactivity in anticancer research, highlighting their potential as leads for antitumor drug development [63].

## 4. Integrated Prevention and Control of *Penicillium*

**Figure 2:**
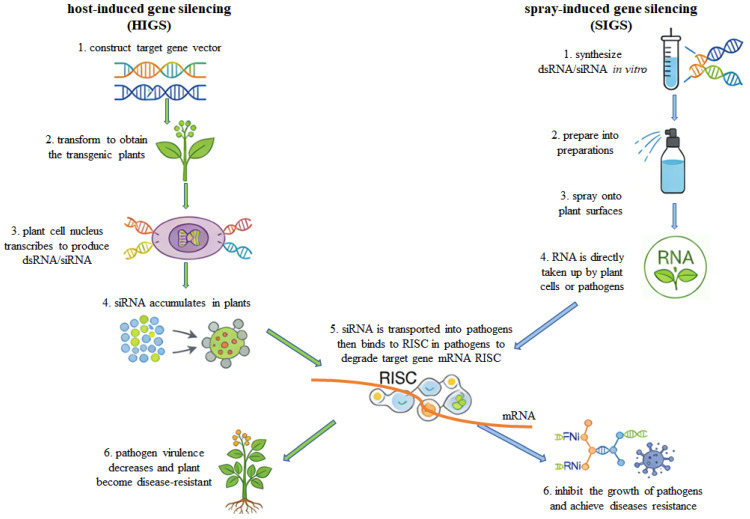
Host-induced gene silencing and spray-induced gene silencing.

Fungal diseases account for a majority of all plant biotic diseases, posing a severe threat to global crop production and food security. These devastating fungal diseases cause up to 14% crop yield losses annually [64]. Traditional chemical control methods are increasingly problematic due to the development of fungicide resistance and concerns about environmental pollution. Conventional breeding approaches for disease resistance are often time-consuming, while the commercial deployment of transgenic technologies faces regulatory and public acceptance challenges. RNAi-based gene silencing technologies have emerged as promising alternatives for sustainable management of fungal diseases and mycotoxin contamination [16,65]. Among these, host-induced gene silencing (HIGS) and spray-induced gene silencing (SIGS) represent the most rapidly advancing ecological friendly control strategies [66]. The fundamental principles underlying these technologies are illustrated in Figure 2.

### 4.1. Host-Induced Gene Silencing (HIGS)

HIGS is an RNAi-based technology that involves introducing dsRNA complementary to pathogen target genes into host plants through methods such as viral vectors or ATMT. Once inside the plant, the dsRNA is processed by plant endonucleases into small interfering RNAs (siRNAs), which are then loaded into the RNA-induced silencing complex (RISC). Upon pathogen infection, these siRNAs are taken up by the invading pathogen and specifically silence the corresponding target genes, thereby inhibiting pathogen growth and infection [67]. This approach not only enables functional validation of pathogen genes in reverse genetics but also facilitates the development of stably inherited disease-resistant crop varieties (Figure 2).

The concept of HIGS was first introduced in 2010 by Nowara and colleagues, who demonstrated gene silencing mediated by the *Barley stripe mosaic virus* [68]. Since then, HIGS has been widely applied in studying disease resistance in crops such as wheat and cotton. Successful applications include the control of wheat leaf rust [69], wheat stripe rust [70], cotton *Verticillium* wilt [71], and banana *Fusarium* wilt [72]. For instance, HIGS targeting the *PtCYC1* [69] and *PsFUZ7* [70] genes in the wheat stripe rust pathogen (*Puccinia striiformis* f. sp. tritici) has resulted in stable, highly resistant wheat lines. Similarly, transgenic cotton plants silencing the *VdH1* gene of *Verticillium dahliae* have shown strong resistance against *Verticillium* wilt [73]. Progress has also been made in developing resistant germplasm against rice blast (*Pyricularia oryzae*) [74] and rapeseed stem rot (*Sclerotinia sclerotiorum*) [75], significantly enriching the available pool of resistance genes.

Despite its potential, HIGS technology has several limitations: (1) applicability is currently limited to crop species with established genetic transformation systems, and the breeding process is often time-consuming; (2) genetic stability and off-target effects can result from random integration of vectors into the plant genome that may lead to unintended phenotypic alterations and silencing non-target genes with sequence homology; (3) target selection requires identifying highly effective target genes in pathogens, which remains challenging, and the technology may not achieve optimal results in some host–pathogen interaction systems; (4) regulatory and ethical concerns remain as HIGS involves the creation of genetically modified organisms (GMOs), its application is subject to stringent regulatory oversight and public acceptance issues, which may limit its commercial deployment.

### 4.2. Spray-Induced Gene Silencing (SIGS)

SIGS is a non-transgenic strategy developed from the HIGS. It involves spraying dsRNAs or siRNA targeting pathogen virulence genes directly onto plant surfaces. Gene silencing is triggered either through plant uptake and transport of the RNA molecules or by direct pathogen ingestion [76]. The underlying mechanism relies on cross-kingdom RNAi, where extracellular vesicles and other pathways facilitate nucleic acid transfer between plants and pathogens (Figure 2). By avoiding genetic modification, SIGS circumvents regulatory and ethical concerns associated with transgenic crops while offering shorter development cycles. Since its introduction, SIGS has demonstrated significant potential for controlling plant diseases in various crops, culminating in the U.S. Environmental Protection Agency (EPA) approving the first sprayable RNA-based biopesticide in 2023 targeting Colorado potato beetle *Leptinotarsa decemlineata*, marking its transition toward commercial application [77].

SIGS strategies can be categorized into single-target and multi-target approaches. Single-target applications include spraying dsRNA targeting the *CYP51* gene in *Fusarium* species to inhibit *Fusarium* head blight in barley [78], as well as dsRNA targeting the *FolRDR1* gene in *Fusarium oxysporum* f. sp. *lycopersici* to alleviate symptoms of tomato *Fusarium* wilt [79]. Multi-target approaches, which involve mixing dsRNAs corresponding to multiple key pathogenicity genes, enhance control efficacy. For example, simultaneously silencing the *Chs7*, *Gls*, and *Pkc* genes in *Fusarium graminearum* reduced wheat *Fusarium* head blight lesions by up to 78% [80]. The effectiveness of SIGS has been validated across multiple crops, including wheat, rice, tomato, and cotton, and it can be integrated with chemical fungicides to reduce overall pesticide usage.

Despite its promise, SIGS technology faces several limitations: (1) environmental instability due to the short half-life of naked dsRNA in field environments (approximately 48 h), as it is susceptible to degradation by nucleases, wash-off by rain, and damage from ultraviolet radiation; (2) variable uptake efficiency as pathogens differ significantly in their ability to take up exogenous dsRNA, for instance, *Colletotrichum* species show minimal uptake, while uptake efficiency in oomycetes varies depending on cell type and developmental stage [81]; (3) unclear molecular mechanisms govern siRNA transport and interaction with Argonaute proteins, and specificity determinants remain incompletely understood; (4) target selection challenges also inhibit progress as identifying highly effective target genes remains difficult, requiring a balance between specificity against target pathogens and broad-spectrum activity against multiple pathogens or isolates.

### 4.3. Technological Improvements, Enhancements, and Regulation

Both HIGS and SIGS face several challenges, including off-target effects and the potential for pathogens to develop resistance. The mechanisms underlying both approaches are regulated by complex RNAi networks. Key processes—such as the transfer of siRNAs between host and pathogen and the role of silencing suppressors—require further investigation. Combining HIGS and SIGS may offer complementary advantages: the rapid action of SIGS together with the sustained resistance provided by HIGS could lead to the development of more effective and long-term disease management strategies. Additionally, exploiting the cross-kingdom regulatory functions of plant endogenous or exogenous microRNAs presents a promising avenue for designing novel HIGS approaches with enhanced disease resistance [82,83].

To enhance the dsRNA stability and delivery efficiency, researchers have developed various delivery systems. Nanocarriers—such as chitosan, carbon quantum dots, and layered double hydroxides—can protect dsRNAs from degradation, improve cellular uptake, and extend the duration of effectiveness [84,85]. Protein-based spherical nanoparticles derived from plant viruses enable efficient dsRNA encapsulation and soil delivery, achieving sustained gene silencing in nematodes by protecting dsRNA from environmental degradation [86]. Food-derived carriers (e.g., grapefruit juice-derived lipids) [87] and beneficial microorganism-based carriers (e.g., *Trichoderma* spp.) [88] offer advantages in terms of safety and cost-effectiveness. Furthermore, *E. coli* expression systems enable large-scale production of dsRNA, reducing manufacturing costs and laying the foundation for industrial application [89,90,91]. Yin and colleagues successfully engineered the RNase III enzyme in *E. coli* to cleave dsRNA into 22–23 bp siRNAs, further advancing RNAi efficiency [92].

Recent field trials have also validated the environmental safety of RNAi crops through comprehensive non-target organism assessments: transgenic cotton expressing dsRNA targeting the FAR gene of the pest *Adelphocoris suturalis* showed no adverse effects on the predatory lady beetle *Harmonia axyridis* across multiple generations, with no detectable trophic transfer of dsRNA through the plant-pest-natural enemy food chain [93]. In plant pathology, SIGS has progressed to advanced field trials targeting fungal pathogens. Notably, researchers have developed self-assembling triangular RNA nanoparticles (Bc-triangle) targeting four virulence genes of *Botrytis cinerea*, demonstrating superior persistence and efficacy compared to linear dsRNA, with lesion area reduction sustained up to 10 days post-spraying in planta [94]. This RNA nanotechnology approach eliminates the need for synthetic nanocarriers, addressing biocompatibility and environmental persistence concerns. Beyond agriculture, RNAi therapeutics have achieved remarkable clinical milestones: SGB-3908, an siRNA medicine targeting angiotensinogen mRNA for hypertension, demonstrated >95% sustained target suppression and durable blood pressure reduction for up to six months after single-dose administration in Phase 1 trials [95], while ARO-ALK7 has entered clinical studies as the first investigational RNAi therapeutic targeting adipose tissue for obesity treatment [96].

Future research should prioritize the development of low-cost, environmentally friendly nanocarriers and delivery technologies—such as pH-responsive nanopesticide systems and biomimetic nanovesicles—to enhance dsRNA stability and delivery efficiency. Optimization of large-scale dsRNA production processes will also be essential to reduce costs and promote industrial application. In parallel, integrating big data and intelligent technologies to establish RNAi target gene screening systems will facilitate the identification of conserved pathogenicity genes, enabling the development of broad-spectrum nucleic acid-based pesticides. Multi-target design strategies can help mitigate the risk of resistance and improve control efficacy against complex diseases.

The international regulatory landscape for HIGS remains fragmented and complex, creating significant hurdles for commercialization. Regulatory bodies like the EPA in the United States and EFSA in Europe mandate thorough biosafety assessments focusing on potential risks including off-target effects on non-target organisms, unintended epigenetic changes, and environmental persistence of dsRNA molecules [97]. A key regulatory challenge stems from the classification of HIGS plants—whether they should be regulated as GMOs with all associated restrictions, which varies considerably across jurisdictions. In Latin America, some countries are developing specific frameworks for RNAi-based technologies, but international harmonization is lacking [98]. Additionally, the variability in HIGS efficacy due to poor understanding of trans-kingdom RNA translocation mechanisms complicates risk assessment and regulatory approval [99].

### 4.4. Biocontrol Agents for Postharvest Penicillium Diseases

Recent advances in biocontrol have diversified the arsenal against postharvest *Penicillium* diseases, encompassing microbial antagonists, actinobacterium-derived metabolites, plant-derived compounds [100], and endophytic bacteria with multifunctional mechanisms. Actinobacterium-derived metabolites have emerged as potent biocontrol agents. *Streptomyces netropsis* DT02 demonstrates strong antifungal activity against *P. italicum*, the causal agent of blue mold in citrus fruits, through production of diverse secondary metabolites including pimprinine, amphotericin B, pimprinethine, N-acetylaureothamine, prothracarcin, and aureothin. These metabolites exhibit inhibition zones ranging from 15 to 45 mm in vitro, creating opportunities for sustainable postharvest disease management in citrus crops [101]. Plant-derived compounds also show remarkable efficacy against *Penicillium* pathogens. Perillaldehyde (PAE), extracted from *Perilla frutescens*, exhibits excellent inhibitory activity against *P. digitatum* with minimum inhibitory concentration and minimum fungicidal concentration of 0.625 mL L^−1^ and 1.25 mL L^−1^, respectively. The compound’s antifungal mechanism involves disruption of cell membrane permeability and integrity, leading to leakage of cellular contents, reactive oxygen species accumulation, and lipid peroxidation. Metabolomic analysis revealed that PAE primarily affects alpha-linolenic acid metabolism, steroid degradation, and steroid hormone biosynthesis pathways, ultimately suppressing mycelial growth and spore germination. In vivo experiments successfully confirmed that PAE reduces lesion diameter and decay rate in citrus during storage, positioning it as a promising natural antifungal agent [102].

Endophytic bacteria represent another innovative biocontrol strategy. *Bacillus amyloliquefaciens* LJ1, isolated from Nanguo pear, exhibits a unique dual intervention mechanism against *P. expansum*. In vitro experiments demonstrated significant inhibition of spore germination, while in vivo studies showed a 66.66% reduction in disease incidence in Nanguo pears after 11 days of storage. Transcriptomic analysis revealed that LJ1 upregulates NAD(P)H-dependent reductase and cytochrome P450 genes, initiating patulin degradation through epoxidation modification. Simultaneously, activated ABC transporters facilitate toxin excretion. Importantly, LJ1 suppresses patulin biosynthesis by upregulating the carbon metabolism repressor gene *PeCreA* and downregulating PAT biosynthesis genes (*PePatG* through *PePatK*). This represents the first confirmation of a dual intervention mechanism involving both patulin synthesis regulation and direct detoxification [103]. Yeast antagonists continue to be extensively studied for citrus biocontrol. Recent research highlights their mechanisms of action, including oxidative burst of reactive oxygen species, iron depletion, and production of secondary metabolites. Emerging approaches such as CRISPR/Cas9, RNAi, and omics technologies are advancing our understanding of yeast-pathogen interactions. Future directions emphasize the development of beneficial microbial consortia comprising core microbial species that metabolically complement each other in interacting networks, potentially offering enhanced efficacy compared to single-strain applications [104]. Bacterial biocontrol agents have demonstrated broad-spectrum activity. *Bacillus* species, including *B. subtilis*, *B. velezensis*, and *B. amyloliquefaciens*, effectively control various postharvest pathogens through volatile organic compound production and nutrient competition, reducing disease incidence by 60–85%. *Pseudomonas fluorescens* and other pseudomonads contribute to disease suppression through similar mechanisms [105]. The integration of multiple biocontrol strategies with physical methods enhances overall efficacy. These integrated approaches represent the future direction of sustainable postharvest disease management.

## 5. Climate Change Impacts on *Penicillium* Distribution and Disease Dynamics

Climate change is fundamentally reshaping the ecological dynamics of postharvest fungal pathogens, with profound implications for *Penicillium* distribution, virulence, and mycotoxin contamination risks [106]. Geographic distribution shifts represent a primary concern under changing climatic conditions. Rising global temperatures and altered precipitation patterns are expanding the geographical range of postharvest pathogens, potentially introducing warm-adapted *Penicillium* species to new regions. This geographic expansion threatens agricultural systems previously unexposed to specific pathogens, requiring adaptive management strategies and heightened surveillance in vulnerable areas. Temperature effects on pathogen biology are multifaceted. Warmer conditions accelerate fungal growth rates and may select for thermotolerant strains with enhanced competitive advantages. Temperature fluctuations during storage and transport directly influence pathogen virulence, sporulation capacity, and infection efficiency. Studies indicate that climate-induced temperature changes can modify the expression of pathogenicity factors and secondary metabolite gene clusters, potentially enhancing disease severity [107]. Humidity and precipitation patterns critically affect postharvest disease dynamics. Increased humidity and extreme weather events disrupt traditional postharvest drying and storage practices, creating favorable conditions for fungal proliferation. Higher moisture content in stored commodities promotes spore germination and mycelial growth, so compromised drying protocols increase contamination risks throughout the supply chain [106].

Mycotoxin production exhibits climate sensitivity, with significant food safety implications. Climate variables influence mycotoxin biosynthesis pathways, potentially increasing toxin accumulation in contaminated commodities. The global mycotoxin survey reveals that climate variability has expanded the ecological niche of toxigenic fungi, with fusariotoxins showing increased prevalence across Europe, Central and South America [108]. While this survey focused on *Fusarium* toxins, similar climate-driven patterns are likely for *Penicillium* mycotoxins, warranting increased monitoring and risk assessment.

Host–pathogen interactions are modified by climate-induced stress in crops. Plants experiencing drought, heat stress, or nutrient imbalances may exhibit weakened natural defenses, making them more susceptible to postharvest infections. This stress-mediated susceptibility creates feedback loops wherein climate-stressed crops become more vulnerable, while simultaneously, warming conditions enhance pathogen fitness. Fungicide resistance development may be accelerated by climate change. Environmental stress can select for resistant genotypes, while altered growing seasons and expanded pathogen ranges increase fungicide application frequency, intensifying selection pressure for resistance. The emergence of multi-drug resistant *Penicillium* strains in major production regions underscores this concern [109].

Adaptive strategies for climate-resilient postharvest management include several approaches. Advanced storage technologies, such as hermetic systems and controlled atmospheres, provide physical barriers against fungal invasion under variable environmental conditions. Predictive modeling tools integrating climate data enable early disease detection and targeted interventions. Region-specific biocontrol formulations that perform consistently across temperature and humidity gradients are essential for sustainable protection. Integration of climate science with agronomy and microbiology will be crucial for developing resilient postharvest management systems that safeguard food supplies in a warming world. The convergence of climate change impacts with evolving pathogen biology necessitates proactive, multidisciplinary approaches combining predictive modeling, adaptive storage technologies, and climate-resilient biocontrol strategies to ensure global food security and safety [110].

## 6. Artificial Intelligence and Future Perspectives

Postharvest diseases caused by *Penicillium* species involve complex, multi-stage biochemical processes. Effective control strategies must therefore be grounded in a thorough understanding of disease development and shift toward sustainable, integrated approaches that prioritize ecological safety, resource efficiency, and consumer health. RNAi-based gene silencing technologies, including HIGS and SIGS, offer efficient, specific, and sustainable solutions for the control of these fungal diseases. Significant progress has been made in applying these approaches to manage various crop diseases. Despite current challenges related to stability, delivery efficiency, and cost, ongoing innovations in vector design, target selection, technological integration, and supportive policy frameworks are expected to drive the widespread adoption of RNAi-based methods [67]. These technologies hold great potential to substantially reduce reliance on chemical fungicides and emerge as key tools for ensuring global food security and advancing sustainable agricultural development. Future efforts should focus on deepening fundamental research into underlying mechanisms, accelerating industrial translation, addressing technical barriers, and expanding application scenarios. Such advances will facilitate the transformation and upgrading of plant disease management strategies.

Although artificial intelligence (AI) technologies have not yet been widely applied to *Penicillium* research specifically, some laboratories have already taken advantage of this transformative approach for the precise identification, efficient fermentation, and risk management of *Penicillium* species, significantly enhancing research efficiency and translational applications. Concurrently, deep learning like RNAsmol now enable accurate prediction of RNA–small molecule interactions using only sequence data, facilitating in silico target selection without requiring structural information [111]. In the field of species identification, deep learning models can automatically extract features such as colony morphology, textural characteristics, and molecular sequence data from *Penicillium* cultures. Recent advances in computer vision have demonstrated that convolutional neural networks and vision transformers can achieve >88% validation accuracy in identifying pathogenic fungi based solely on colony architecture, with some models reaching 93.9% accuracy for fungal classification using microscopic images [112]. This enables high-precision, high-throughput identification at the genus and species levels, with some systems achieving high accuracy rates representing a substantial improvement over traditional manual microscopy and conventional taxonomic methods [113,114].

Beyond identification, machine learning algorithms are increasingly being integrated with multi-omics approaches [115] to predict antifungal resistance, model host–pathogen interactions, and optimize fermentation parameters [116]. For fermentation control, deep reinforcement learning already has been applied to optimize batch management in *Penicillium* fermentation processes, allowing for real-time optimization of critical parameters such as temperature and pH, thereby improving both the yield and quality stability of penicillin production. Traditional fermentation methods rely heavily on empirical manual adjustments and lack precise control [117]. Furthermore, AI can be integrated with sensor technologies to enable real-time monitoring and localization of *Penicillium* contamination providing an efficient and scalable technological foundation for contamination control and management. There is no doubt that AI will becoming increasing important in guiding *Penicillium*-related research in the near future, particularly as portable sequencing technologies and field-deployable diagnostic tools become more accessible [116]. The translation of RNAi technology from laboratory research to commercial applications has accelerated dramatically, with several high-profile field trials and regulatory approvals demonstrating its practical potential. These developments are supported by advanced bioinformatics tools such as dsRNAmax, which uses machine learning to design highly specific dsRNA sequences that minimize off-target effects on beneficial organisms while maximizing efficacy against target pests [118]. Together, these advances underscore the maturation of RNAi technology and AI-assisted decision-making across medical and agricultural domains.

## Figures and Tables

**Table 1:** The main known pathogenic genes of *Penicillium* spp. and their functions.

Penicillium	Gene	Protein	Role and Mechanism in Pathogenicity	Control Strategies
*P. digitatum*	*PacC*	pH signaling response transcription factor	This gene plays a global regulatory role in mediating pathogen adaptation to the host microenvironmental pH. Its deletion results in complete loss of pathogenicity [20].	Fungicide, microbial antagonists, natural plant-derived products, HIGS, SIGS
	*PdChsVII*	chitin synthase	This gene is involved in cell wall biogenesis and maintains cell wall integrity. Its deletion leads to growth defects and reduced pathogenicity [21].	
	*Pmt2*	protein O-mannose transferase	This gene is involved in the glycosylation of cell wall proteins and contributes to maintaining cell wall integrity. Its deletion affects fungal growth, sporulation, and sensitivity to antifungal peptides [22].	
	*Sca*	cysteine-rich anion secretion protein	This gene produces a protein that lacks direct antimicrobial activity but effectively counteracts the effects of host-derived or exogenously applied antifungal proteins (such as AfpB), thereby significantly enhancing infection success [23].	
	*FlbC*	transcription factor	This gene positively regulates resistance to demethylation inhibitor (DMI) fungicides, such as imazalil. Its deletion results in hypersensitivity without altering ergosterol levels, indicating that it regulates a novel resistance pathway independent of the target enzyme [24].	
	*PdSreA/B*	sterol regulatory element-binding protein (SREBP) homolog	This gene influences the sensitivity of fungal strains to DMI fungicides by regulating the expression of multiple sterol biosynthesis genes, including *CYP51* [25].	
	*PdMpkB*	MAPK kinase (Fus3/Kss1 homology)	The mutant strain is nearly incapable of causing fruit rot, because of downregulated expression of multiple cell wall-degrading enzyme genes [26].	
	*PdMfs1/PdMfs2*	major facilitator superfamily (MFS) transporter	This gene may be involved in the secretion of toxic compounds. Mutants exhibit reduced virulence on citrus fruits [27].	
*P. expansum*	*PePatA-PePatO*	patulin biosynthesis gene cluster	The patulin biosynthetic gene cluster comprises 15 genes, among which *PePatL* and *PePatK* play key roles in patulin biosynthesis [19].	HIGS, SIGS, plant volatile compounds, the mixture and heterogeneous fungicide
	*PeSte12*	transcription factor	This gene regulates hyphal fusion and host penetration, thereby influencing pathogenicity [28].	
	*PeStuA*	APSES family transcription factor	This gene globally regulates hyphal growth, asexual sporulation, pathogenicity, and patulin biosynthesis [29].	
	*PepatA*	acetate transporter	This gene positively regulates sporulation and patulin accumulation by modulating acetate metabolism [30].	
*P. italicum*	*PiCaMK1*	a new calcium/calmodulin-dependent protein kinase	This gene regulates multiple physical and cellular processes including growth, conidiation, virulence, and environmental stress tolerance [31].	HIGS, SIGS, fungicide
	*SntB*	the epigenetic reader	Its deletion leads to the significant phenotypic alterations, including delayed mycelial growth, reduced spore production, and decreased utilization of sucrose, and it also increases sensitivity to pH and reduces the virulence [32].	
	*Piwsc1*	a cell wall integrity-related gene	Its deletion reduces virulence on citrus fruits, and decreases the growth rate of mycelia, the germination rate of spores [33].	

## Data Availability

The original contributions presented in this study are included in the article. Further inquiries can be directed to the corresponding author.
